# ATF3 Modulates the Endoplasmic Reticulum Stress-Induced Impairment of Milk Synthesis in Bovine Mammary Epithelial Cells

**DOI:** 10.3390/ijms27104250

**Published:** 2026-05-10

**Authors:** Chen Zhang, Wenting Dai, Yue Liu, Hongwei Xu, Hongyun Liu

**Affiliations:** 1 College of Animal Sciences, Zhejiang University, Hangzhou 310058, China; zchenn@163.com (C.Z.); liuyue19418@163.com (Y.L.); 2 College of Pharmaceutical Sciences, Zhejiang University of Technology, Hangzhou 310014, China; daiwenting@zjut.edu.cn; 3 School of Bioengineering, Northwest Minzu University, Lanzhou 730030, China; xuhongwei@xbmu.edu.cn

**Keywords:** activating transcription factor 3, endoplasmic reticulum stress, milk synthesis, apoptosis, bovine mammary epithelial cells

## Abstract

Due to the substantial secretory burden, bovine mammary epithelial cells (BMECs) are highly susceptible to endoplasmic reticulum (ER) stress caused by the accumulation of misfolded proteins when protein-folding capacity is overwhelmed. However, how ATF3 regulates ER stress-induced impairment of milk synthesis and apoptosis in BMECs, particularly through its direct transcriptional targets, remains poorly understood. In this study, we investigated the protective role of activating transcription factor 3 (ATF3) against ER stress-induced impairment of milk synthesis in BMECs. Using a tunicamycin-induced ER stress model, we overexpressed *ATF3* in BMECs and performed integrated RNA-seq and ChIP-seq analyses to elucidate the underlying molecular mechanisms. Our results indicated that ER stress disrupted milk protein and fat synthesis in BMECs by suppressing the expression of CSN2, FASN, FABP3 and promoting apoptosis via upregulation of BAX and CASP3. *ATF3* overexpression effectively attenuated these effects, reducing apoptosis and restoring the expression of milk fat-related genes. Transcriptomics demonstrated that ATF3 activated MAPK and PI3K-Akt signaling and lipid metabolism pathways, significantly upregulating key genes involved in fatty acid uptake, transport, and metabolism (*CD36*, *SLC27A1*, *ACSL1*, *PLIN1*). Integrated RNA-seq and ChIP-seq analyses identified 81 overlapping genes, with *RASGRP2*, *PRKACB*, *MAP3K5*, and *DUSP10* confirmed as direct transcriptional targets of ATF3, mediating its regulation of the MAPK pathway. Collectively, these findings elucidate the protective role of ATF3 against ER stress-induced lactation disruption and offer potential molecular targets for enhancing lactation resilience in dairy cattle under stress.

## 1. Introduction

The mammary gland serves as a critical organ for milk synthesis and secretion. Mammary epithelial cells (MECs) exhibit high metabolic activity during lactation [1,2]. MECs are highly susceptible to endoplasmic reticulum (ER) stress due to the increased protein production required during late pregnancy and lactation [3]. This secretory demand places a substantial burden on the ER, the central organelle responsible for protein folding and maturation [4]. When the protein-folding capacity of the ER is exceeded, unfolded or misfolded proteins accumulate in the ER lumen, thereby triggering ER stress and activating the unfolded protein response (UPR) [5]. The UPR initially functions as an adaptive mechanism to restore proteostasis by enhancing protein folding, attenuating mRNA translation, and inducing apoptosis if homeostasis is not restored [6].

In the mammary gland, maintenance of ER homeostasis is essential for the survival and secretory function of epithelial cells during lactation [2]. Persistent ER stress may compromise epithelial cell viability, impair the synthesis of milk components, and ultimately reduce lactation performance in vivo [7]. In dairy cows, BMECs are exposed to multiple physiological and pathological stressors during lactation, including fatty acid overload, hypoxia, heat stress, inflammation, and oxidative imbalance [8,9,10,11]. Notably, it has been shown that fatty acid overload in ketotic cows induces ER stress in BMECs, leading to apoptosis and decreased milk synthesis [12]. These findings suggest that ER stress may act as a critical molecular link between adverse metabolic or environmental conditions and lactation dysfunction.

Activating transcription factor 3 (ATF3) is a stress-inducible transcription factor that has been implicated in the regulation of adaptive responses, and metabolic homeostasis [13], suggesting that it may serve as an important molecular regulator of cellular stress adaptation [14,15]. In our unpublished single-cell multi-omics analysis, we identified a BMEC subpopulation with elevated heat-shock protein expression and enrichment of ER stress-related pathways, accompanied by increased transcriptional activity of ATF3. These findings suggested that ATF3 may participate in the stress-responsive regulatory network of BMECs and prompted us to further investigate the functional role of ATF3 in ER stress-induced impairment of milk synthesis.

Although previous studies have investigated ER stress in BMECs [16], the molecular mechanisms linking this stress to lactation deficits and apoptosis remain unclear. In particular, the transcriptional regulators that coordinate stress adaptation and cellular fate in BMECs have not been fully characterized. To establish an in vitro model of ER stress, we used tunicamycin (Tm), a classical pharmacological inducer, to induce ER stress in BMECs [17,18]. Therefore, the present study aimed to investigate the role of ATF3 in the regulation of ER stress-induced dysfunction in BMECs, with a particular focus on milk synthesis and apoptosis. This study may provide new insights into the molecular mechanisms underlying ER stress-induced dysfunction in BMECs.

## 2. Results

### 2.1. Effects of Endoplasmic Reticulum Stress on the Viability of BMECs and Their Unfolded Protein Response

**Figure 1:**
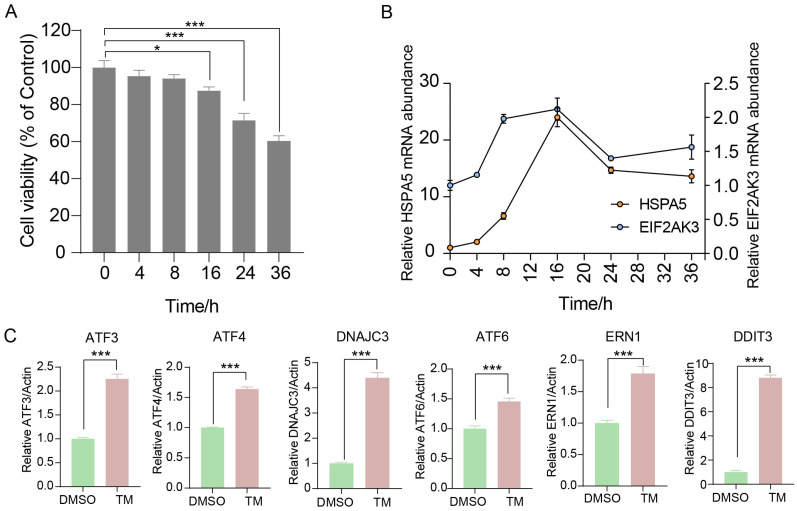
Effects of endoplasmic reticulum stress on cell viability and the unfolded protein response in BMECs. (**A**): Cell viability (% of Control) of BMECs under ER stress at 0, 4, 8, 16, 24, and 36 h. (**B**): A 36 h time course of *HSPA5* and *EIF2AK3* mRNA abundance under ER stress. (**C**): The mRNA abundance of UPR-related genes (*ATF4*, *ATF3*, *ATF6*, *DNAJC3*, *ERN1* and *DDIT3*) in BMECs under ER stress. Data with error bars represent mean ± SEM. Statistical significance (*** *p* < 0.001, * *p* < 0.05) was determined by unpaired *t*-test.

Treatment with 0.5 μg/mL tunicamycin (Tm) for 12 h significantly reduced BMEC viability compared with the control group (Figure S1). To further determine the appropriate treatment duration, BMECs were treated with 0.5 μg/mL Tm for different durations (0, 4, 8, 16, 24 and 36 h). Cell viability was significantly decreased after 16 h of Tm treatment (Figure 1A). The mRNA expression of the ER stress markers *HSPA5* and *EIF2AK3* were maximally induced at 16 h after Tm treatment and decreased substantially after 24 h (Figure 1B). Based on these results, 0.5 μg/mL Tm for 16 h was selected for subsequent experiments. In addition, Tm treatment induced a marked increase in the mRNA expression of ER stress-related genes, including *ATF3*, activating transcription factor 4 (*ATF4*), activating transcription factor 6 (*ATF6*), DnaJ heat shock protein family [Hsp40] member C3 (*DNAJC3*), endoplasmic reticulum to nucleus signaling 1 (*ERN1*), and DNA damage inducible transcript 3 (*DDIT3*) (Figure 1C).

### 2.2. Effects of Endoplasmic Reticulum Stress on Milk Synthesis and Apoptosis in BMECs

**Figure 2:**
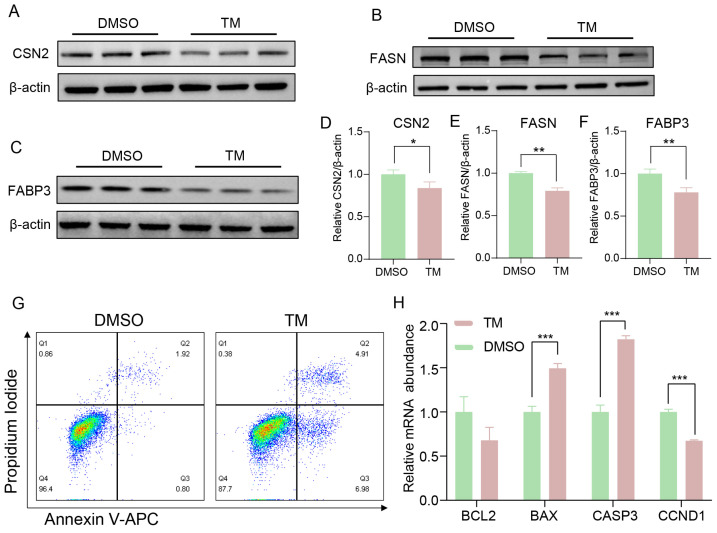
Effects of endoplasmic reticulum stress on milk synthesis in BMECs. (**A**–**F**): Immunoblots and quantitative analyses of CSN2, FASN and FABP3. (**G**): Flow cytometry analysis of apoptosis (Annexin V-APC/PI) in BMECs under ER stress. (**H**): The relative mRNA abundance of apoptosis-related genes in BMECs under ER stress. Data with error bars represent mean ± SEM. Statistical significance (*** *p* < 0.001, ** *p* < 0.01, * *p* < 0.05) was determined by unpaired *t*-test.

ER stress significantly reduced the protein expression of CSN2 (Figure 2A,D), FASN (Figure 2B,E), and FABP3 (Figure 2C,F) in BMECs. In addition, ER stress increased apoptosis in BMECs (Figure 2G). The mRNA expression of the pro-apoptotic genes *BAX* and *CASP3* was upregulated, whereas *CCND1* was downregulated (Figure 2H). Furthermore, mitochondria were elongated and structurally disorganized (Figure S2A). The mRNA expression of mitochondrial dynamics (*OPA1*, *MFN2*, *FIS1*, and *ATP5F1*) were significantly reduced (Figure S2B).

### 2.3. Effects of ATF3 on Milk Synthesis and Apoptosis in BMECs

**Figure 3:**
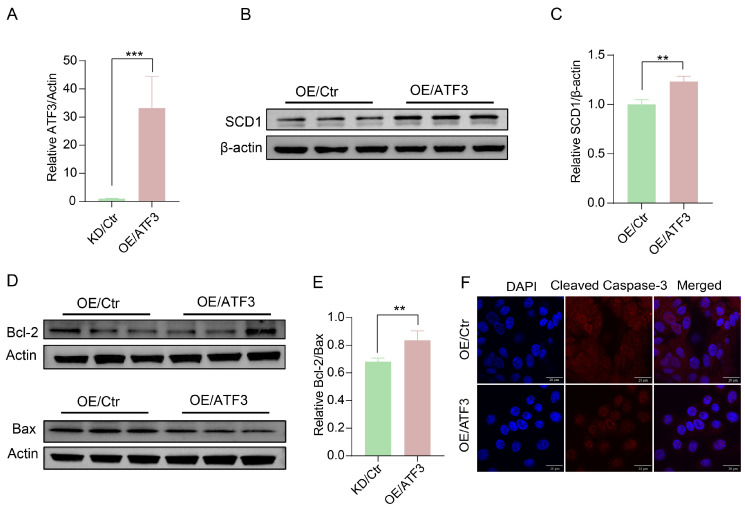
Effects of *ATF3* overexpression on milk fat synthesis and apoptosis in BMECs. (**A**): *ATF3* mRNA abundance in BMECs transduced with *ATF3*-overexpressing lentiviral vectors (OE/ATF3) or empty vector controls (OE/Ctrl). (**B**,**C**): Immunoblots and quantitative analyses of Stearoyl-Coenzyme A desaturase-1 (SCD1) in BMECs transduced with *ATF3*-overexpressing lentiviral vectors (OE/ATF3) or empty vector controls (OE/Ctrl). (**D**,**E**): Immunoblots and quantitative analyses of Bax and Bcl-2 in BMECs. (**F**): Immunofluorescence staining for cleaved caspase-3 in BMECs. DAPI for nuclei (blue), scale bar: 20 μm. Data with error bars represent mean ± SEM. Statistical significance (*** *p* < 0.001, ** *p* < 0.01) was determined by unpaired *t*-test.

The *ATF3* was overexpressed in BMECs via lentiviral transduction and overexpression efficiency was confirmed by qRT-PCR (Figure 3A). *ATF3* overexpression increased milk fat synthesis-related genes (*LPIN2*, *PPARGC1B*, *ACSL4*) (Figure S4) and SCD1 protein expression (Figure 3B,C)in BMECs and reduced apoptosis (Figure 3D). Bax protein expression was decreased, whereas the Bcl-2/Bax ratio was increased (Figure 3E). (Figure 3E). Immunofluorescence analysis showed reduced cleaved caspase-3 expression in *ATF3*-overexpressing cells (Figure 3F). In contrast, *ATF3* knockdown significantly decreased the mRNA expression of genes involved in milk fat synthesis (*LPIN2*, *CPT1A*, *PPARGC1B*, *ACSL4*, *ACOX2*) but had no effect on genes related to milk protein synthesis (Figure S3D,E).

### 2.4. Effect of ATF3 Overexpression on the Transcriptome of BMECs

**Figure 4:**
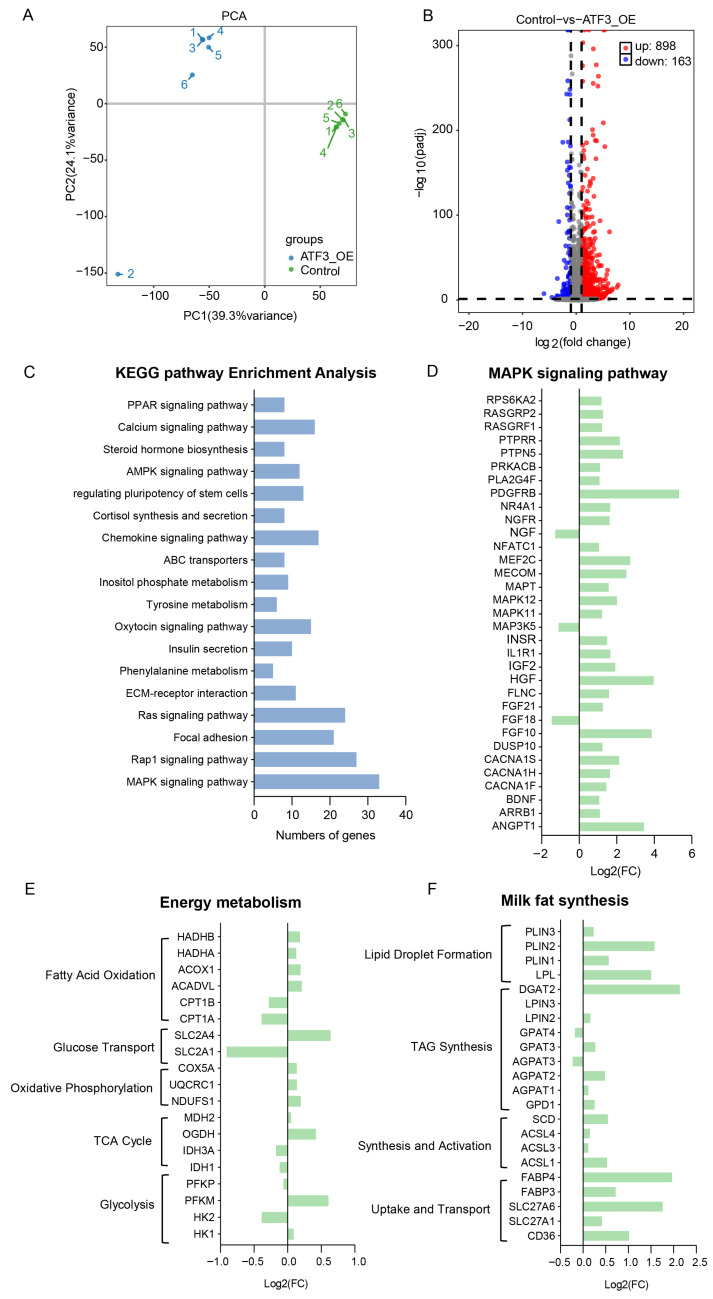
Effects of *ATF3* overexpression on the transcriptome of BMECs. (**A**): Principal component analysis (PCA) of BMECs transcriptomes. Each point is one biological replicate (*n* = 6); colors indicate treatment (OE/ATF3, blue; OE/Ctrl, green). (**B**): Volcano plot of differentially expressed genes (DEGs) in ATF3-OE. The vertical dotted lines indicate the fold-change threshold, and the horizontal dotted line indicates the statistical significance threshold. (**C**): KEGG pathway enrichment of DEGs from OE/ATF3. Bar length indicates the number of genes. (**D**): MAPK signaling pathway related-genes among the DEGs. (**E**): Energy metabolism-related genes. The genes were classified glycolysis (*HK1*; *HK2*; *PFKM*; *PFKP*), TCA Cycle (*IDH1*; *IDH3A*; *OGDH*; *MDH2*), oxidative phosphorylation (*NDUFS1*; *UQCRC1*; *COX5A*), glucose transport (*SLC2A1*; *SLC2A4*), fatty acid oxidation (*CPT1A*; *CPT1B*; *ACADVL*; *ACOX1*; *HADHA*; *HADHB*). (**F**): Milk fat synthesis-related genes. The genes were classified into uptake and transport of FA (*CD36*; *SLC27A1*; *SLC27A6*; *FABP3*; *FABP4*), activation and synthesis of FA (*ACSL1*; *ACSL3*; *ACSL4*; *SCD*, Stearoyl-CoA desaturase 1), TAG synthesis (*GPD1*; *AGPAT1*; *AGPAT2*; *GPAT3*; *LPIN1*; *LPIN2*; *LPIN3*; *DGAT2*), Lipid droplet secretion (*PLIN1*; *PLIN2*; *PLIN3*; *LPL*).

Principal component analysis (PCA) showed clear separation between the OE/ATF3 and OE/Ctrl groups (Figure 4A). In total, 1061 differentially expressed genes (DEGs) were identified in the OE/ATF3 group, of which 898 were upregulated and 163 were downregulated (Figure 4B). KEGG pathway analysis indicated that these DEGs were significantly enriched in the MAPK, Rap1, focal adhesion, and PPAR signaling pathways (Figure 4C). In the MAPK signaling pathway, *FGF18*, *MAP3K5*, and *NGF* were downregulated, whereas most other genes were upregulated (Figure 4D). Genes involved in glycolysis, the tricarboxylic acid cycle, oxidative phosphorylation, glucose transport, and fatty acid oxidation also showed altered expression (Figure 4E). Genes involved in fatty acid uptake, transport, activation, and synthesis, as well as lipid droplet secretion (e.g., *CD36*, *SLC27A1*, *SLC27A6*, *ACSL1*, *ACSL3*, *PLIN1*, and *PLIN2*) were upregulated (Figure 4F).

### 2.5. Effects of ATF3 on Target Gene Expression in BMECs

**Figure 5:**
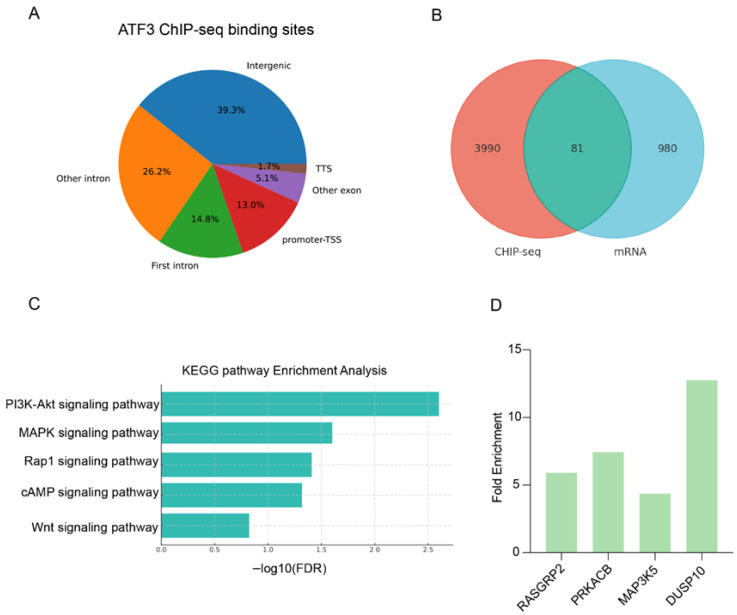
Genomic analyses of ATF3 downstream targets in BMECs. (**A**): Genomic distribution of ATF3 ChIP-seq peaks across annotations (Promoter-TSS, exon, intron, TTS, intergenic). (**B**): Venn diagram showing the overlap between ATF3-bound genes (ChIP-seq peak–associated genes) and differentially expressed genes (DEGs) from the RNA-seq analysis; numbers indicate gene counts. (**C**): KEGG enrichment of the overlap genes (*n* = 81). (**D**): *RASGRP2*, *PRKACB*, *MAP3K5*, and *DUSP10* were selected as the downstream target gene of ATF3. Bars are ordered by adjusted *p* value (FDR); bar length represents −log10 (FDR).

ChIP-seq analysis showed that 13.0% of ATF3-binding sites were located in the promoter-transcription start site (TSS) region, 14.8% in the first intron, 26.2% in other introns, 39.3% in intergenic regions, 1.7% in the transcription termination site (TTS), and 5.1% in other exons (Figure 5A). A total of 81 overlapping genes were identified by integrating the ChIP-seq and RNA-seq datasets (Figure 5B). KEGG pathway analysis showed that these genes were enriched in the MAPK, PI3K-Akt, Rap1, cAMP, and Wnt signaling pathways (Figure 5C). Integrated ChIP-seq and RNA-seq analysis revealed that *RASGRP2*, *PRKACB*, *MAP3K5*, and *DUSP10*, which are associated with the MAPK signaling pathway, were identified as direct target genes of ATF3 (Figure 5D).

## 3. Discussion

During lactation, the mammary epithelium sustains high rates of protein and fat synthesis, making it vulnerable to proteostatic imbalance and ER stress. Although ER stress in the mammary gland of mice has been extensively studied, the molecular coupling of stress signals to lactation deficits and apoptotic responses in BMECs remains unclear. Our analysis revealed that ER stress disrupts milk fat and protein synthesis and induces apoptosis. We also demonstrated that ATF3 regulates these processes through MAPK signaling and lactation-related gene networks, highlighting its critical role in maintaining BMEC homeostasis under ER stress.

Tm-induced ER stress in BMECs upregulated *ATF4* and *DDIT3* expression, promoting cell death, consistent with their pro-apoptotic roles [19]. We observed the upregulation of *BAX* and *CASP3*, along with the downregulation of *CCND1*, which is in line with previous findings of apoptosis in secretory epithelial cells under stress [20]. Furthermore, ER stress led to mitochondrial elongation and structural disorganization, along with reduced expression of genes related to mitochondrial dynamics (*MFN2*, *OPA1*, and *FIS1*), suggesting disrupted ER-mitochondria crosstalk under stress [21]. Mitochondrial elongation may reflect ER stress-induced fusion-fission imbalance, amplifying UPR-induced apoptosis via ATF4 and DDIT3 activities [22]. OPA1 and MFN2 are critical for mitochondrial fusion, maintaining organelle integrity, and efficient ATP production [23], whereas FIS1 regulates fission to balance mitochondrial turnover [24]. The coordinated downregulation of these genes may reflect defective bioenergetic adaptation during ER stress [25]. Together with the reduction in ATP5F1-a (a key subunit of mitochondrial ATP synthase), these changes indicate that ER stress not only activated cell death pathways but also decreased expression of milk synthesis genes (e.g., CSN2 and FASN) under ER stress.

The downregulation of milk fat (FASN and FABP3) and milk protein (CSN2) synthesis genes following tunicamycin treatment suggests that tunicamycin-induced ER stress disrupts the core metabolic and secretory machinery responsible for milk synthesis, potentially through protein misfolding or translational inhibition [6,26]. These results support previous studies indicating that ER stress may impair milk synthesis during lactation in dairy cow [27]. Our findings suggest that unresolved ER stress may directly compromise the milk-synthesizing capacity of the mammary epithelium, thereby impairing milk yield and quality. From a pathophysiological perspective, the combined suppression of milk protein and milk fat synthesis, together with increased epithelial apoptosis and mitochondrial dysfunction, implies not merely an acute cellular stress response, but a progressive loss of functional milk-secreting capacity. Such alterations would be expected to reduce the number and activity of secretory epithelial cells, thereby destabilizing mammary gland homeostasis and ultimately contributing to lower milk yield and altered milk composition under stress-related conditions [28]. In dairy cows, this mechanism may be particularly relevant in metabolic challenges such as negative energy balance, ketosis, and heat stress, all of which have been increasingly associated with ER stress-related impairment of mammary epithelial function [29].

ATF3 is a stress-responsive transcription factor induced by diverse cellular insults, including ER stress, oxidative stress, and cytokine exposure [13,30]. Our findings showed that *ATF3* was induced in BMECs under ER stress, and its overexpression decreased apoptosis, aligning with its cytoprotective role [31]. This anti-apoptotic effect is further supported by a study showing that endothelial-specific ATF3 loss in pulmonary vessels increased apoptosis and decreased proliferation [32]. Notably, ATF3 also promotes survival in immune cells by enhancing macrophage survival and proliferation [33]. These findings are consistent with our results, collectively underscoring the conserved function of ATF3 in promoting cellular adaptation and survival across diverse cell types. In our model, *ATF3* overexpression also increased the expression of several milk fat synthesis-related genes, including *CD36*, *SLC27A1*, *SLC27A6*, *ACSL1*, *ACSL3*, *PLIN1*, and *PLIN2*, indicating ATF3 not only attenuates cell death but also helps preserve the metabolic program required for lactation. These findings suggest that ATF3 may function as an adaptive regulator that buffers the detrimental effects of ER stress on both epithelial survival and milk-synthetic competence [34,35]. Transcriptomic profiling of the *ATF3*-overexpressing BMECs revealed significant enrichment of the DEGs in the MAPK, Rap1, focal adhesion, and PPAR signaling pathways. The upregulation of genes related to the MAPK signaling pathway and fat synthesis underscores the role of ATF3 in coordinating pro-survival signaling with metabolic reprogramming to sustain fat synthesis during stress adaptation [30,36].

Integrated ChIP-seq and RNA-seq analyses identified 81 overlapping genes, with KEGG pathway enrichment highlighting the MAPK, PI3K-Akt, Rap1, cAMP, and Wnt signaling pathways. These pathways play crucial roles in cell-fate decisions, where a balance between proliferation, apoptosis, and metabolic adaptation is vital for sustaining lactation under stress. Notably, the MAPK and PI3K-Akt pathways are well-established mediators of cellular survival and apoptotic resistance in mammary epithelial cells [37,38]. In our study, ChIP-seq analyses showed that ATF3 directly regulates the MAPK pathway genes RASGRP2, PRKACB, MAP3K5, and DUSP10, confirming these as downstream targets under its transcriptional control. *ATF3* overexpression in BMECs decreased the expression of MAP3K5 while upregulating DUSP10, suggesting a coordinated repression of pro-apoptotic signaling via the MAPK signaling pathway [39,40]. Additionally, *ATF3* overexpression increased RASGRP2 and PRKACB expression, linking ATF3 to ERK signaling and supporting survival under stress [41,42]. These findings suggest that ATF3 could be a molecular target for mitigating stress-induced lactation losses and improving milk yield and quality in dairy cattle.

While these findings provide important insights into the role of ATF3 in ER stress adaptation and milk synthesis in BMECs, several aspects should be taken into consideration. The present findings were primarily derived from an in vitro BMEC model, and further validation in mammary organoids and in vivo systems will be necessary to better reflect the mammary microenvironment and lactation physiology. Further studies addressing these aspects will strengthen the mechanistic understanding of ATF3 function and may offer new insights into improving mammary health and lactation performance in dairy cows.

## 4. Materials and Methods

### 4.1. Sample and Ethics Aspects

BMECs were isolated and cultured as described previously [43,44]. The use of animal tissues was approved by the Institutional Animal Use Committee of Zhejiang University (ZJU2017-0718). Briefly, mammary gland tissues were obtained aseptically from mid-lactation Holstein dairy cows. After washing with PBS and D-Hanks solution, the tissues were cut into small pieces with sterile surgical scissors and digested sequentially with trypsin and collagenase I/II at 37 °C. The digested suspension was filtered through sterile gauze and a 70 μm cell strainer, followed by centrifugation to collect cells. The isolated cells were cultured in Dulbecco’s modified Eagle’s medium (DMEM)/F12 supplemented with 5 μg/mL transferrin (T0665, Sigma, St. Louis, MO, USA), 5 μg/mL insulin (I8040, Solarbio, Beijing, China), 1 μg/mL hydrocortisone (G8450, Solarbio, Beijing, China), 10 ng/mL epidermal growth factor (E4127, Sigma, St. Louis, MO, USA), 1% penicillin-streptomycin (CP011, Keyi, Hangzhou, China), and 10% fetal bovine serum (10099141, Gibco, Grand Island, NY, USA) at 37 °C in a humidified incubator with 5% CO_2_. BMECs were purified from fibroblasts based on their differential sensitivity to trypsin digestion, and cells at passages 3–4 were used for subsequent experiments.

### 4.2. Cell Culture

Cells were cultured in a complete medium consisting of Dulbecco’s modified Eagle’s medium (DMEM)-F12 supplemented with 5 μg/mL transferrin, 5 μg/mL insulin, 1 μg/mL hydrocortisone, 10 ng/mL epidermal growth factor, 1% (*w*/*v*) penicillin-streptomycin, and 10% (*w*/*v*) fetal bovine serum in a humidified atmosphere containing 5% (*v*/*v*) CO_2_. Tunicamycin was dissolved in DMSO to prepare a stock solution and then diluted in culture medium to different concentrations. BMECs were cultured in a humidified incubator [37 °C, 5% (*v*/*v*) CO_2_, 95% (*v*/*v*) air] for 24–36 h before treatment. After replacing the medium with fresh culture medium, BMECs were treated with tunicamycin at different concentrations (0, 0.5, 1, 2, 5, and 10 μg/mL) or for different durations (0, 4, 8, 16, 24, and 36 h). Cell viability was then assessed using a CCK-8 assay.

### 4.3. Cell Viability and Apoptosis Detection

Cell viability was assessed using the CCK-8 assay (C0038; Beyotime, Shanghai, China), following tunicamycin treatment. Absorbance was measured at 450 nm using a microplate reader (Tecan Spark, San Jose, CA, USA). Apoptosis was analyzed by flow cytometry using the Annexin V-APC/PI apoptosis kit (Elabscience, Wuhan, China), according to the manufacturer’s protocol.

### 4.4. Immunoblot

BMECs were lysed on ice in RIPA buffer (P0013B, Beyotime, Shanghai, China) containing a 1× protease-phosphatase inhibitor mixture (P1045, Beyotime, Shanghai, China). Total protein content was determined using a BCA protein assay kit (P0009, Beyotime, Shanghai, China). Proteins were resolved by SDS-PAGE and transferred to PVDF membranes (IPVH00010, Sigma, St. Louis, MO, USA). The antibodies used for this study were listed in Table S1. Signals were captured using a chemiluminescence imaging system (CLiNX Science Instrument, Shanghai, China) and quantified with ImageJ software v1.53 (National Institutes of Health, Bethesda, MD, USA). Relative protein abundance was normalized to β-actin.

### 4.5. Quantitative Real-Time PCR Assay

Total RNA (1 µg) was reverse-transcribed using HiScript III RT Super Mix for qPCR kit (R323, Vazyme, Nanjing, China). The cDNA was used for real-time PCR with Pro Universal SYBR qPCR Master Mix and specific primers (Table S2) on a 7500 Real-Time PCR System (Applied Biosystems, Carlsbad, CA, USA). β-Actin (ACTB) was used as the internal reference for normalization, and relative mRNA abundance was calculated using the 2^−∆∆CT^ approach.

### 4.6. Triglyceride Content Measurement

Triglyceride content was measured using a commercially available assay kit (E1013, Applygen, Beijing, China). BMECs were lysed, and supernatants were incubated with chromogenic reagent at 70 °C. Absorbance at 550 nm was measured, and triglyceride content was normalized to total protein concentration.

### 4.7. Small Interfering RNA Transfection

After reaching 50% confluence, BMECs were transfected with negative control (NC) siRNA or custom-designed *ATF3* siRNA (Tsingke Biotechnology Co., Ltd., Beijing, China) using Lipofectamine™ RNAiMAX Transfection Reagent (13778150, Invitrogen, Waltham, MA, USA), according to the manufacturer’s instructions. The *ATF3* siRNA sequences were 5′-CGAGAAGCAGCAUUUGAUA-3′ (sense) and 5′-UAUCAAAUGCUGCUUCUCG-3′ (antisense). After 6 h of transfection, the medium was replaced with complete medium, and the cells were cultured for an additional 12 h. Subsequently, the cells were treated with tunicamycin and incubated at 37 °C for another 16 h.

### 4.8. Lentiviral Vector Construction and RNA-Seq of BMECs

The coding sequence of *ATF3* from Bos taurus (GenBank accession number: NM_001046193.2) was cloned into the lentiviral expression vector. BMECs were transduced with either the *ATF3* overexpression lentiviral vector or the control vector. After stable overexpression was established, both groups were treated with tunicamycin under the indicated conditions to induce ER stress (*n* = 6). Total RNA was isolated from cultured cells using Trizol reagent (Invitrogen) in accordance with the manufacturer’s guidelines. Sequencing was conducted on the Illumina NovaSeq™ 6000 platform. Genes were designated as differentially expressed if they met the criteria of |log_2_(fold change)| ≥ 1 and *p* < 0.05.

### 4.9. ChIP-Seq Library Preparation and Analysis

Cells were crosslinked with 1% formaldehyde for 10 min at room temperature and quenched with 125 mM glycine. The fragmented chromatin fragments were pre-cleared and then immunoprecipitated with Protein A + G Magnetic beads coupled with anti-Flag (F1804, Sigma-Aldrich, St. Louis, MO, USA) antibodies. The DNA libraries were amplified for 15 cycles and sequenced using Illumina NovaSeq 6000 (Illumina, San Diego, CA, USA). The reads were aligned to the bovine reference genome (Bos taurus ARS-UCD1.2), and alignments were sorted and stored via SAMtools (v1.10). Peak calling was performed with MACS2 (v2.2.7.1). Functional annotation of genes associated with significant peaks was conducted using clusterProfiler v4.0 for KEGG pathway enrichment.

### 4.10. Immunofluorescence Staining

Cells were seeded on coverslips in 24-well plates. At ~60% confluence, cells were washed twice with ice-cold PBS and fixed with a cell fixative at room temperature for 10 min. After PBS washes, cells were permeabilized for 5–10 min, blocked for 1 h, and incubated with primary antibody at 4 °C overnight. Cells were then incubated with fluorescent secondary antibody for 1 h in the dark and counterstained with DAPI for 1 min. Coverslips were mounted using an antifade mounting medium to minimize fluorescence quenching, and images were acquired using an Olympus IX81-FV3000 laser confocal microscope (Olympus, Tokyo, Japan).

### 4.11. Mitochondrial Ultrastructure Analysis

BMECs were cultured in 6 cm dishes until reaching 90% confluence. Cells were collected, washed twice with PBS, and fixed in 2.5% glutaraldehyde at 4 °C for 4 h. The samples were then post-fixed with osmium tetroxide and uranyl acetate, dehydrated through a graded ethanol series, embedded, and sectioned for transmission electron microscopy. Images were acquired using a Talos 120 kV cryo-transmission electron microscope (Thermo Fisher Scientific, Waltham, MA, USA).

### 4.12. Statistics Analysis

Cellular experiment data were visualized and visualized using Graphpad Prism (Boston, MA, USA; Version 10.2) software. Sequencing data were analyzed and visualized in R version 4.2.0 (Foundation for Statistical Computing, Vienna, Austria). Data were presented as mean ± SEM. All data were normally distributed. Statistical comparisons were performed using a two-tailed unpaired *t*-test, with *p* < 0.05 considered statistically significant.

## 5. Conclusions

In summary, ER stress impairs milk synthesis in BMECs by downregulating the expression of milk fat and protein genes, increasing apoptosis, and disrupting mitochondrial structure and function. ATF3, a stress-inducible transcription factor, suppresses apoptosis, enhances fat synthesis via MAPK signaling, and regulates lactation-related genes in BMECs. These findings offer novel insights that could be helpful for developing strategies to mitigate stress-induced lactation losses and improve milk production in dairy cows.

## Data Availability

The datasets produced and/or analyzed during the current study are available from the corresponding author on reasonable request.

## Supplementary Materials

The following supporting information can be downloaded at: https://www.mdpi.com/article/10.3390/ijms27104250/s1.
