# Supplementary material for: ATF3 Modulates the Endoplasmic Reticulum Stress-Induced Impairment of Milk Synthesis in Bovine Mammary Epithelial Cells
Source: Int J Mol Sci. 2026 May 10;27(10):4250. doi: 10.3390/ijms27104250 (PMC13206812; doi:10.3390/ijms27104250)
Supplement: Supplementary file 1 [file ijms-27-04250-s001.zip › ijms-4302727-supplementary.pdf]

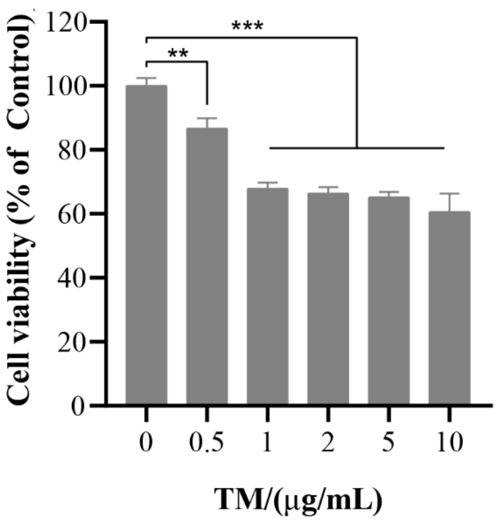

Figure S1. Effects of different concentrations of tunicamycin (Tm, ER stress inducer) on the cell viability (% of Control) of BMECs after 12 h of treatment. Data with error bars represent mean ± SEM. Statistical significance (****P* < 0.001, ***P* < 0.01) was determined by unpaired t-test.

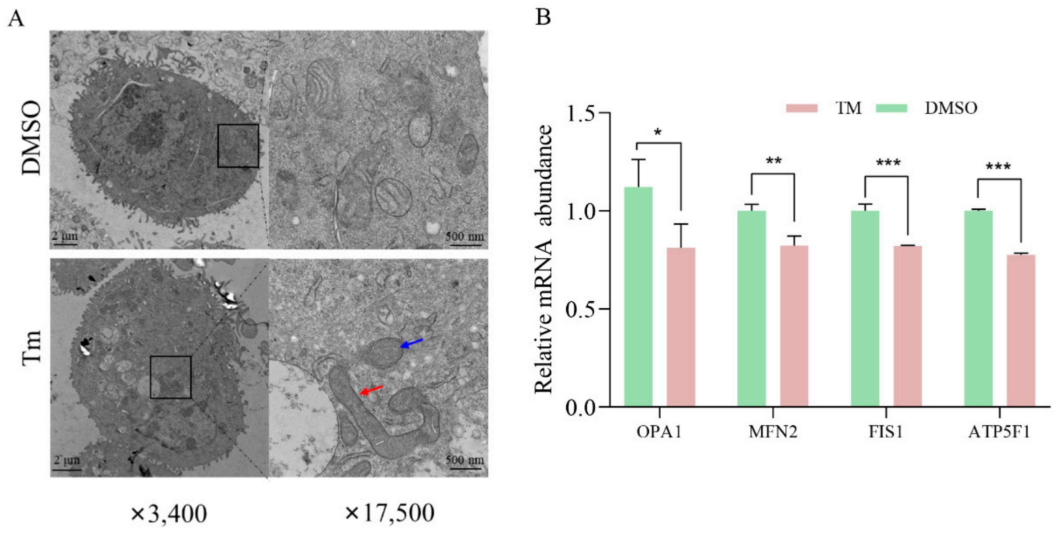

Figure S2. Effects of endoplasmic reticulum stress on mitochondrial homeostasis in BMECs. **A**: The mitochondrial structure and function of BMECs under ER stress. **B**: Relative mRNA expression of mitochondrial homeostasis-related genes in BMECs under ER stress. Data with error bars represent mean ± SEM. Statistical significance (****P* < 0.001, ***P* < 0.01, **P* < 0.05) was determined by unpaired t-test.

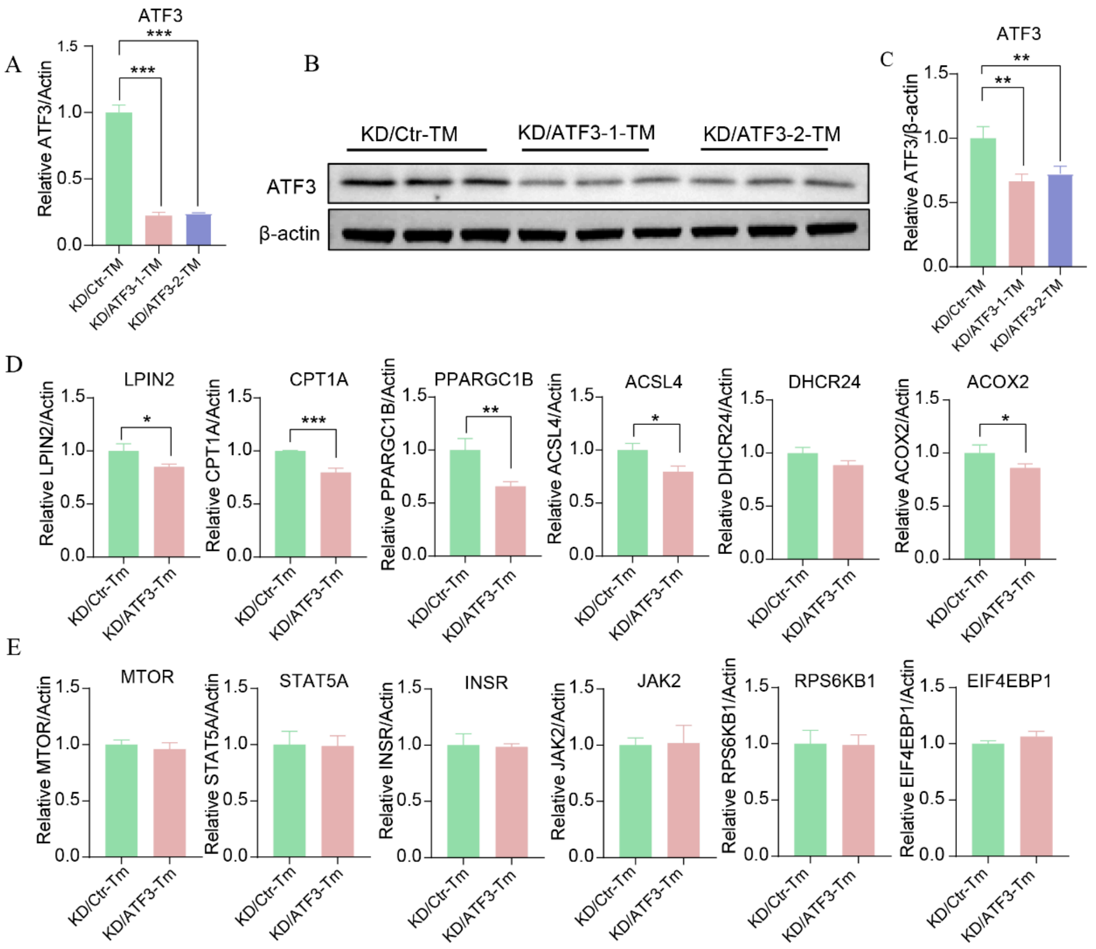

Figure S3. Effects of ATF3 knockdown on milk synthesis in BMECs under ER stress. A: Relative mRNA abundance of ATF3 in BMECs transfected with either the control (NC) siRNA or ATF3 siRNA. **B-C**: Immunoblots and quantitative analyses of ATF3 in BMECs with ATF3 knockdown. **D-E**: Relative mRNA abundance of milk fat (**D**) and protein (**E**) synthesis-related genes in BMECs with ATF3 knockdown. Data with error bars represent mean ± SEM. Statistical significance (****P* < 0.001, ***P* < 0.01, **P* < 0.05) was determined by unpaired t-test.

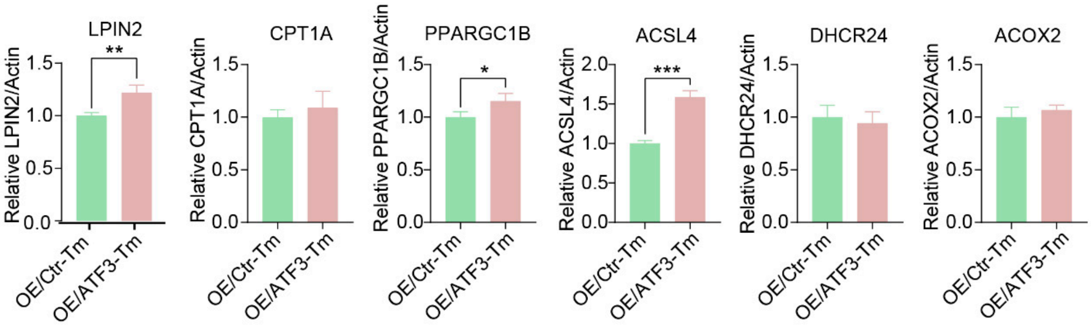

Figure S4. Relative mRNA abundance of ATF3 overexpression on milk fat synthesis in BMECs under ER stress. Data with error bars represent mean ± SEM. Statistical significance (****P* < 0.001, ***P* < 0.01, **P* < 0.05) was determined by unpaired t-test.

Table S1. Antibodies for immunoblot

Protein	Supplier	Item Number
CSN2	GeneTex (Irvine, CA, USA)	66115
FASN	Abcam (Cambridge, UK)	ab128870
FABP3	Abcam (Cambridge, UK)	ab124978
Bax	Beyotime (Shanghai, China)	AB026
Bcl2	Beyotime (Shanghai, China)	AB112
β-Actin	Affinity Biosciences (Jiangsu, China)	T0022
Goat anti-mouse IgG (H+L), HRP	Affinity Biosciences (Jiangsu, China)	S0002
Goat anti-rabbit IgG (H+L), HRP	Affinity Biosciences (Jiangsu, China)	S0001

Table S2. Primers used for quantitative real-time PCR

Gene symbol	NCBI Reference Sequence	Forward/ Reverse	Primer sequences (5'-3')	Amplification length (bp)
ACOX2	NM_001102015.2	F	GCATGGGACAGAGGCTGAG	191
		R	ATGCATGCTACTGCTCCAGG	
ACSL4	XM_015461586.3	F	CTGCTGCGGCTTTTTCTCCG	149
		R	TCTGGATTTGAATCCGAGGCG	
ACTB	NM_173979.3	F	GTGACAGCAGTCGGTTGGAT	151
		R	GTGGCTTTTGGGAAGGCAAA	
ATF3	NM_001046193.2	F	ACTGCATAGCGCTCACTCT	186
		R	GTTGAAGCATCATTTTGCTCCTG	
ATF4	NM_001034342.2	F	CCCTTCGACCAGTTGGGTTT	180
		R	GGAGAAAGCATCCTCCTTGC	
ATF6	XM_024989876.2	F	AGCCCTGATGGTGCTAACTGA	100
		R	TTCATGATTTAACCTGAGAGATTCTGTT	
ATP5F1	NM_001038501.2	F	GGGGCGGGAAGATTGCTG	127
		R	TTGCCTGCAATACCCCTGG	
BAX	NM_173894.1	F	CCCTTTTGCTTCAGGGTTTCA	120
		R	TCAGACACTCGCTCAGCTTC	
BCL2	NM_001166486.1	F	TCATGTGTGTGGAGAGCGTC	134
		R	CTCCACAAAGGCGTCCCAG	
CASP3	NM_001354777.2	F	CTGAGGGTCAGCTCCTAGCG	171
		R	TGCTTCCATGTAAGATCTTTGTCTC	
CCND1	NM_001046273.2	F	ATCAGATGTGACCCGGACTG	180
		R	TCAGATGTTCACGTCACGCA	
CPT1A	NM_001304989.2	F	GCACCTCTGTCCACAGACTC	192
		R	CCGGTGATGATGCCATTCTTG	
DDIT3	NM_001078163.1	F	CCCTGGAAACAAGGAGGAAGAAT	144
		R	AGGGAGCTCTGACTGGACTC	
DHCR24	NM_001103276.1	F	CCACAACGACATCCACGTCT	194
		R	CGTGCACACTTCGGACAAAC	
DNAJC3	NM_174756.3	F	CCTCTCCGATCCAGAAATGAGG	126
		R	GGACTGAACCCTTGCCATGA	
EIF2AK3	NM_001098086.2	F	GCCGTGCGGCAGATCATTA	99
		R	ACGTCCAAATCCCACTGCTT	
EIF4EBP1	NM_001077893.2	F	TGGAGTGTCGGAACTCACCT	162
		R	ACTGTGACTCTTCACCGCC	
ERN1	XM_024980955.2	F	AACACACGTGGAAGAGCCTG	150
		R	ATGCCATCTGAACTTCGGCA	
FIS1	NM_001034784.2	F	ACAGAGCCACAGAACAAC	142
		R	CAGCAAGTCCGATGAGTC	

HSPA5	NM_001075148.1	F	CGTGCGTTTGAGAGCTCAGT	98
		R	CCAGTCGGTCAGCAGTCAG	
INSR	XM_005208817.5	F	GTTTTCATCCCCAGGCCTTC	171
		R	GACCAGCGACTCCTTGTTCA	
JAK2	XM_024996127.2	F	GCACCGGGTTTCAGAAGCA	100
		R	ACAGGTGTGATTCCTTCGGG	
LPIN2	XM_059881123.1	F	TCTGAAACATCCAGAACCTCTCA	101
		R	GCCTGGTTGATGCCCTTGTA	
MFN2	NM_001190269.1	F	GGCATTCTCGTTGTTGGA	169
		R	GCTTCTCACTGGCATACTC	
MTOR	NM_001386500.1	F	CTTAGAGGACAGCGGGGAAG	111
		R	TCCTTTAATATTCGCGCGGC	
OPA1	NM_001192961.1	F	TCTTCAGGCTCGTCTCAA	187
		R	CTCATCACTCGCAAGGTAA	
PPARGC1B	XM_005209630.5	F	GACTTGGAGCTCTTCCAGAAATG	104
		R	TGATCTAGGGTCAATCTTCCCTCT	
RPS6KB1	NM_205816.1	F	TATGCCTTTCAGACCGGTGG	125
		R	TCTGCCAAGTAAAAGCAGGC	
STAT5A	NM_001012673.1	F	AACATGTACCCACAGAACCCTG	182
		R	ATTCAGGACAGCGAGCCTCT	
